# A Simple New Visualization of Exercise Data Discloses Pathophysiology and Severity of Heart Failure

**DOI:** 10.1161/JAHA.112.001883

**Published:** 2012-06-22

**Authors:** James E. Hansen, Xing-Guo Sun, William W. Stringer

**Affiliations:** St. John's Cardiovascular Research Center, Los Angeles Biomedical Research Institute at Harbor–UCLA Medical Center, Division of Respiratory Physiology and Medicine, Department of Medicine, University of California at Los Angeles David Geffen School of MedicineTorrance, CA

**Keywords:** cardiac output, exercise, heart failure, oxygen, ventilation

## Abstract

**Background:**

The complexity of cardiopulmonary exercise testing data and their displays tends to make assessment of patients, including those with heart failure, time consuming.

**Methods and Results:**

We postulated that a new single display that uses concurrent values of oxygen uptake / ventilation versus carbon dioxide output / ventilation ratios (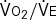
–versus–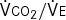), plotted on equal X–Y axes, would better quantify normality and heart failure severity and would clarify pathophysiology. Consecutive 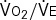
–versus–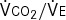 values from rest to recovery were displayed on X–Y axes for patients with Class II and IV heart failure and for healthy subjects without heart failure. The displays revealed distinctive patterns for each group, reflecting sequential changes in cardiac output, arterial and mixed venous O_2_ and CO_2_ content differences, and ventilation (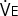). On the basis of exercise tests of 417 healthy subjects, reference formulas for highest 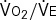 and 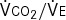, which normally occur during moderate exercise, are presented. Absolute and percent predicted values of highest 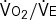 and 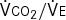 were recorded for 10 individuals from each group: Those of healthy subjects were significantly higher than those of patients with Class II heart failure, and those of patients with Class II heart failure were higher than those of patients with Class IV heart failure. These values differentiated heart failure severity better than peak 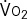, anaerobic threshold, peak oxygen pulse, and 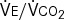 slopes. Resting 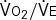
–versus–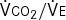 values were strikingly low for patients with Class IV heart failure, and with exercise, increased minimally or even decreased. With regard to the pathophysiology of heart failure, high 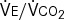 values during milder exercise, previously attributed to ventilatory inefficiency, seem to be caused primarily by reduced cardiac output rather than increased 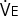.

**Conclusion:**

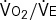
–versus–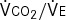 measurements and displays, extractable from future or existing exercise data, separate the 3 groups (healthy subjects, patients with Class II heart failure, and patients with Class IV heart failure) well and confirm the dominant role of low cardiac output rather than excessive 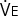 in heart failure pathophysiology. **(J Am Heart Assoc. 2012;1:e001883 doi: 10.1161/JAHA.112.001883.)**

Cardiopulmonary exercise tests are often challenging to interpret because of the magnitude of data collected. Nevertheless, in patients with cardiovascular disease, useful measurements include O_2_ pulse, O_2_ uptake (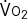), and work rate ratios to assess likelihood of coronary artery disease, plus peak 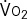 and anaerobic threshold to assess the necessity for heart transplantation^1–4^ or likelihood of severe postoperative complications.^5^ Alone or in combination, the relationship of exhaled ventilation (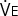) to CO_2_ output (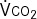),^4–10^ end-tidal gas tensions,^11^ oscillatory breathing,^12^ and the ratio of 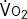 to 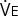 also have been shown to be useful prognosticators of heart failure mortality and morbidity.^5,13,14^ We postulated that displaying some exercise data in a new way would simplify presentation, reveal new information, ease comprehension, and be clinically useful for cardiologists evaluating their patients.

Past explanations of heart failure based on exercise tests primarily have emphasized the findings of high ventilatory inefficiency (high 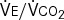 slopes or ratios), high dead space–to–tidal volume ratios, and mismatching of alveolar ventilation–to–cardiac output ratios (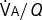).^6,7,15,16^ Unfortunately, in heart failure, the numerator of each of these ratios, by emphasizing the role of the lung and 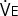, tends to minimize the importance of the heart and cardiac output. Because, by the Fick principle, 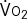 = cardiac output times arterial − mixed venous O_2_ content difference [C(a−v)O_2_] and 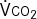 = cardiac output times mixed venous − arterial CO_2_ content difference [C(v−a)CO_2_], we hypothesized that directly using 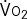 and 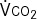 as numerators and 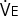 as denominator would better demonstrate the importance of cardiac output at rest and during exercise in both healthy subjects and patients with heart failure. Furthermore, plotting these 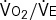
–versus–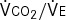 ratios against each other on X–Y axes would best demonstrate the important differences between these changing ratios from rest through exercise to recovery. These displays offered the optimal way to demonstrate that low cardiac output, not high 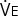, is the dominant factor causing high 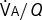, high 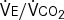, and high dead space–to–tidal volume ratios during ordinary activities in patients with severe left heart failure.

In this series of patients with left heart failure, values of the 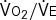
–versus–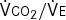 ratios measured from rest to moderate exercise are more discriminating than resting echocardiography, invasive hemodynamics, or other maximal exercise values. Because we could not identify other publications that featured or sequentially displayed concurrent 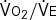
–versus–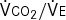 values, we thought it would be useful to share our preliminary data, displays, and knowledge.

## Methods

From past data files of prior Harbor-UCLA publications^10,12,14,17,18^ previously obtained with informed consent and approved by the Harbor-UCLA Institutional Review Board, incremental exercise studies were retrospectively selected. These exercise studies included 10 subjects considered healthy, 10 patients with single diagnoses of New York Heart Association (NYHA) Class II heart failure, and 10 patients with single diagnoses of NYHA Class IV heart failure.

All of the patients with heart failure were participants in St. Jude Medical device studies and were carefully seen, screened, classified, treated, and managed by cardiologists at other institutions. All had systolic dysfunction with left ventricular ejection fractions <35%. None were considered to have significant anemia or pulmonary, neuromuscular, or skeletal disorders.^10,12,14^ Of the 508 patients with heart failure who were available for selection, the vast majority were classified as having NYHA Class III heart failure. Only 16 were classified as having NYHA Class IV heart failure and 52 as having NYHA Class II heart failure. Ten of the 16 patients with Class IV heart failure were randomly selected for comparison with an equal number (n=10) of the 52 patients with Class II heart failure of similar sex, age, and size. For the patients with heart failure, ejection fractions had been obtained by echocardiography and resting hemodynamics by cardiac catheterization within 3 weeks of exercise tests. All patients were considered to be optimally medicated by their physicians; most were receiving β-blockers, diuretics, and angiotensin-converting enzyme inhibitors or angiotensin receptor blockers.

With a physician in attendance, gas exchange and ECG measurements of subjects or patients were made with well-maintained commercial equipment systems (eg, Medical Graphics [St. Paul, MN], SensorMedics [Anaheim, CA], Cosmed [Rome, Italy], or Jaeger [Wurzburg, Germany]) that were properly calibrated just before each test. The protocols required measurements during 3 minutes of rest and 3 minutes of unloaded cycling or comparable warm-up on the treadmill, followed by a progressively increasing work rate in ramp pattern or 1-minute intervals to maximum tolerance, followed by 2 to 3 minutes of recovery. The schedule for increasing the work rate was designed to have the subjects smoothly reach their maximum tolerated work rate in 6 to 15 minutes of exercise, whether on the cycle or treadmill. Whether a cycle or treadmill was used, work rate was increased at 10 to 30 Watts/min for healthy subjects and 5 to 15 Watts/min for patients according to their size, sex, and perceived health. Breath-by-breath data were interpolated to second-by-second values, sequentially averaged in 10-second bins, and plotted traditionally with heart rate data in 9 panels.^17^ Peak 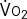 was measured as the highest average 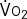 over 30 seconds; peak heart rate and peak O_2_ pulse were measured over that same time period. The anaerobic threshold was measured by the V-slope method.^17^ 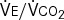 slopes were calculated by regression of concurrent values before ventilatory compensation occurred.

For each exercise test, time, activity level, 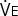 (body temperature pressure saturated), and 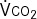 and 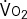 (both standard temperature pressure dry) data were extracted into an Excel (Microsoft Corp) spreadsheet. 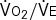 and 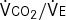 were directly calculated in milliliters per liter for each time period from rest through unloaded cycling, maximally tolerable incremental exercise, and early recovery. With Origin 7 software (OriginLab Corp, Northampton, MA) and colored lines and data points to identify the progression of values, 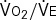
–versus–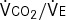 data were graphed sequentially on equal X–Y axes every 10 seconds, plus rolling 30-second, 60-second, and 90-second averages. Tidal volume and ventilatory frequency data were extracted from several cases to ascertain the effect of mouthpiece dead space. Because fluctuations in displays were highest when 10-second and 30-second data were averaged, moderate when 60-second data were averaged, and lowest when 90-second data were averaged, only the latter will be displayed.

Reference mean ± standard deviation (mean±SD) values for 90-second averages of highest 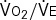 for 417 nonathletic healthy subjects have been reported previously as oxygen uptake efficiency plateau values.^19^ Data from these same subjects^20^ were used to calculate normal reference 90-second values for highest mean±SD 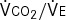. Percent predicted^21^ and mean±SD values of relevant gas exchange variables and parameters were calculated for the 3 previously identified groups. Analyses of variance (with Tukey tests) or unpaired 2-tailed *t* tests were used to compare variables. *P* values <0.05 were considered statistically significant.

## Results

Table 1 gives reference formulas for highest 90-second 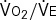 and 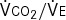 for the 417 healthy subjects. Table 2 gives calculated reference values for ages 30 and 70 years with heights of 160 and 190 cm for men and 150 and 180 cm for women. Normal highest 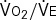 and 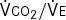 depend dominantly on age and are slightly higher in men and taller individuals. Within these age and height ranges, the lower limits of normal values are 80±2.0% of the predicted mean for highest 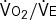 and 83±2.0% of predicted mean for highest 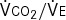. Reducing the duration of measurement averages from 90 seconds to 60 seconds or removing valve dead space 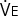 from the measured 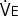 each increases the highest values of 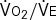 and 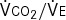 by <1 mL/L.

**Table 1.** Reference Formulas (n=417) for Highest 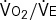 and Highest 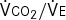

	Constant	Age (y)	Height (cm)	If Female	*P*	RSD
Highest 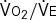 , mL/L	42.2	−0.189	0.036	−3.0	<0.0001	4.62

Highest 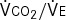 , mL/L	38.9	−0.155	0.038	−1.9	<0.0001	3.72

RSD indicates residual standard deviation; 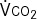, CO_2_ output, standard temperature pressure dry; 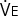, exhaled ventilation, body temperature pressure saturated; and 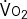, O_2_ uptake, standard temperature pressure dry.

**Table 2.** Normal Values for Highest 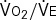 and Highest 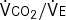

Men (n=281)				

Usual range of ages and heights:				

Age, y	30	30	70	70

Height, cm	190	160	190	160

Predicted values for highest 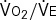	43.4	42.3	35.8	34.7

Predicted values for highest 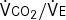	41.4	40.3	35.2	34.1

Women (n=136)				

Usual range of ages and heights:				

Age, y	30	30	70	70

Height, cm	180	150	180	150

Predicted values for highest 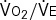	40.0	38.9	32.4	31.3

Predicted values for highest 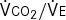	39.2	38.0	33.0	31.8

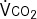
 indicates CO_2_ output, standard temperature pressure dry; 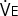, exhaled ventilation, body temperature pressure saturated; and 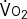, O_2_ uptake, standard temperature pressure dry.

In Table 3 and Table 4, specific *P* value differences between groups are indicated by single or multiple asterisks (*) or daggers (†). Demographics do not differ significantly except for lower ages in the healthy subjects, but the patients with Class IV heart failure show a trend (*P*>.05) toward lower ejection fractions and lower stroke volumes than those seen in the patients with Class II heart failure. The upper half of Table 4 shows multiple significant differences in exercise values among the 3 groups, with the greatest differences seen between the healthy subjects and the patients with Class IV heart failure (*P*<0.05 or 0.01). The lower half of Table 4 is most revealing in several ways. First, the highest 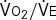 and highest 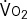 at the same times (obtained during moderate exercise) and the highest 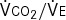 generally are more statistically discriminating among patients with Class IV heart failure, patients with Class II heart failure, and healthy subjects than are the values of peak 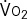, anaerobic threshold, peak O_2_ pulse, or 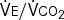 slope. Second, the respectively mean highest 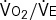 and 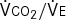 of 28.7 and 25.3 mL/L in patients with Class II heart failure are significantly higher (≈36%) than those of 20.7 and 19.0 mL/L in the patients with Class IV heart failure (*P*<0.001 and *P*<0.01, respectively). In a comparison of the patients with Class II heart failure versus those with Class IV heart failure, the 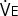 values at these times of highest 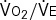 and 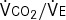 are only minimally (≈11%) and insignificantly (*P*>.05) higher (23.9 versus 21.8 L/min and 26.7 versus 23.9 L/min). Given the numerators, C(a−v)O_2_ and C(v−a)CO_2_ should not be larger in Class II than Class IV heart failure,^22^ so the dominant difference between the groups must be cardiac output. Because 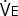 values are only minimally and insignificantly different, this means that low perfusion of the lung (and systemic circulation) rather than excessive 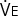 is the dominant cause of the lower 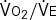 and 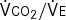 and therefore the higher 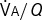, dead space–to–tidal volume ratios, and 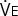
–versus–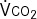 slopes commonly found in Class IV heart failure. Furthermore, at these same times, with only 13% higher 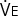 in the healthy subjects than in the patients with Class II heart failure (26.9/23.9=113%), the 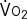 in the healthy subjects is 81% higher (1.07/0.59=181%).

**Table 3.** Demographics and Resting Measurements of Healthy Subjects and Patients With Heart Failure of Different Severities

	Healthy Subjects (n=10)	Patients With NYHA Class II Heart Failure` (n=10)	Patients With NYHA Class IV Heart Failure (n=10)
Sex, male/female	7/3	8/2	7/3

Age, y	56.4±9.9 (38–74)	69.7±9.3*	72.8±10.8 (53–88)*

Height, cm	170.1±8.2 (158–187)	171.1±10.0 (157–191)	169.5±4.8 (163–178)

Weight, kg	79.6±15.6 (54–113)	79.0±16.7 (57–104)	79.1±7.7 (70–92)

Resting measurements			

LVEF, %	…	26.7±6.7 (17–39)	20.8±6.6 (13–37)

SV, mL	…	64.5±13.2 (42–81)	62.5±15.3 (34–81)

Q, L/min	…	4.2±0.9 (3.0–5.5)	4.3±1.1 (2.2–6.6)

CI, L/min per square meter	…	2.2±0.5 (1.6–3.2)	2.2±0.6 (1.1–3.1)

HR, bpm	62.3±5.2 (52–71)	65.2±7.1 (55–77)	69.8±7.8 (59–85)

SBP, mm Hg	126.8±11.2 (109–138)	116±24.9 (90–160)	127.4±202.2 (100–170)

DBP, mm Hg	71.2±9.1 (63–87)	66.8±10.7 (50–82)	74.1±14.9 (50–100)

Data are shown as mean±SD and range (minimum–maximum).

LVEF indicates left ventricular ejection fraction; SV, stroke volume; Q, cardiac output; CI, cardiac output index; HR, heart rate; SBP, systolic blood pressure; and DBP, diastolic blood pressure.

* *P*<0.01, analysis of variance, repeated comparison with healthy subjects. No significant differences between NYHA Classes II and IV.

**Table 4.** Key Measurements of Cardiopulmonary Exercise Testing in Healthy Subjects and Patients With Heart Failure of Different Severities

	Healthy Subjects (n=10)	Patients With NYHA Class II Heart Failure (n=10)`	Patients With NYHA Class IV Heart Failure (n=10)
Peak 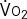			

L/min	1.96±0.56 (1.3−3.1)	1.01±0.31 (0.49−1.45)***	0.62±0.21 (0.36−1.12)****††

mL/min per kilogram	24.8±6.0 (17.4−37.3)	13.2±4.6 (4.9−22.6)***	7.8±2.5 (4.4−13.4)**** ††

% Predicted	95.8±8.6 (81−107)	59.0±20.8 (36−105)***	40.3±15.7 (21−68)**** †

AT			

L/min	1.11±0.29 (0.71−1.51)	0.74±0.14 (0.50−1.00)**	0.50±0.13 (0.40−0.82)***††

mL/min per kilogram	14.0±3.3 (10.5−20.6)	9.9±2.8 (7.5−17.3)*	6.3±1.6 (4.7−9.8)**** ††

% Predicted	98.5±12.8 (79−123)	64.7±23.7 (44−130)**	44.4±7.4 (34−56)**** †

Peak O_2_P			

mL/beat	12.7±3.3 (8.1−18.7)	9.7±4.1 (4.6−16.4)	6.4±1.7 (2.8−8.8)*** †

% Predicted	95.8±8.6 (81−107)	82.4±26.5 (46−123)	61.4±19.2 (24−93)*** †

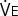 vs 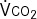			

Slope	25.8±3.8 (22−33)	38.8±9.3 (27−55)**	49.2±11.2 (34−75)**** †

% Predicted	106.9±14.8 (89−131)	141.7±25.6 (105−190)*	163.3±44.8 (106−261)**

Highest 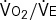			

mL/L	40.8±5.2 (30–47)	28.7±5.3 (21–36)***	20.7±1.6 (18–24)**** †††

% Predicted	100.3±11.3 (71–114)	83.2±15.4 (58–96)**	57.8±4.7 (49–66)**** †††

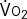 at highest 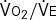 , L/min	1.07±0.30 (0.69–1.49)	0.59±0.18 (0.35–0.95)**	0.42±0.10 (0.25–0.62)**** ††

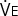 at highest 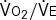 , L/min	26.9±6.2 (19–40)	23.9±6.7 (13–35)	21.8±6.1 (13–33)

Highest 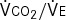			

mL/L	34.6±5.6 (26.2–41.8)	25.3±4.7 (18–35)**	19.0±1.6 (17–22)**** ††

% Predicted	101.4±12.1 (81–117)	73.7±12.2 (53–95)***	56.5±5.3 (47–66)**** ††

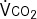 at highest 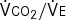 , L/min	0.96±0.17 (0.68–1.21)	0.69±0.15 (0.44–0.94)*	0.47±0.12 (0.39–0.78)**** ††

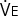 at highest 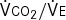 , L/min	28.9±6.2 (22–42)	26.7±5.8 (15–35)	23.9±6.4 (17–38)

Data are shown as mean±SD and range (minimum–maximum).

Peak 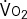 indicates oxygen uptake at maximum exercise; AT, oxygen uptake at anaerobic threshold; O_2_P, oxygen pulse; 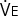 vs 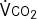, slope of 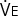 against 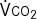; highest 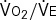
**,** 90-s averaged highest value of ratio of 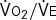; and highest 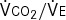
**,** 90-s averaged highest value of ratio of 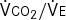.

* *P*<0.05

** *P*<0.01

*** *P*<0.001

**** *P*<0.0001; analysis of variance, repeated comparison with healthy subjects.

† *P*<0.05

†† *P*<0.01

††† *P*<0.001; analysis of variance, repeated comparison with NYHA Class II heart failure.

**Figure 1.**
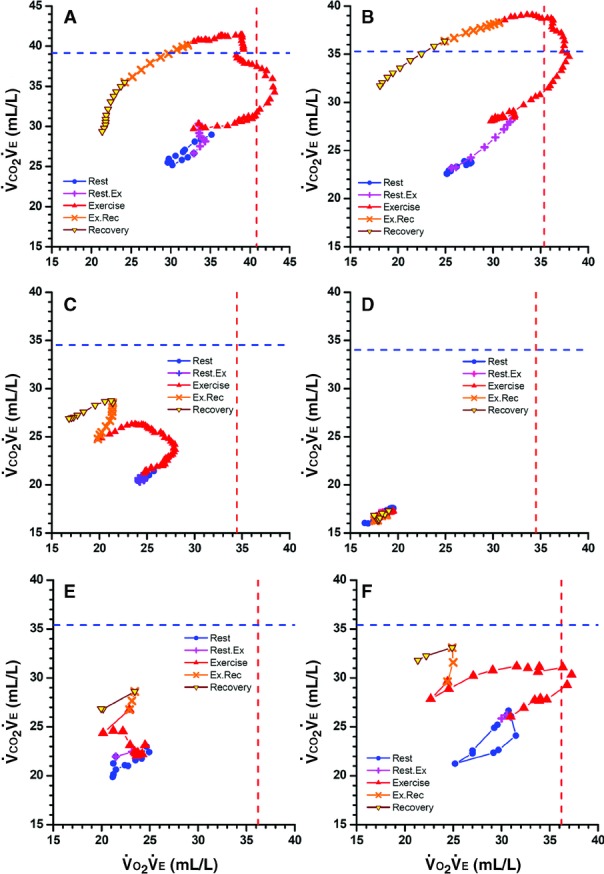
Displays of concurrent 90-s averages of O_2_ uptake and CO_2_ output, both divided by exhaled ventilation (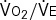
–versus–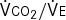) during cardiopulmonary exercise testing from rest through early recovery, with “+” and “×” showing transitions between rest and exercise and exercise and recovery. Vertical and horizontal dashed lines are reference highest 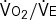 and highest 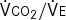. A, Healthy 38-year-old man with peak 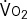 106% of predicted. B, Healthy 52-year-old woman with peak 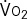 101% of predicted. Note that with exercise, both numerators increase more than denominators until reaching highest 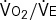 and then highest 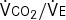 before rapid declines, especially in 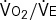 values, during high-intensity exercise and recovery. C, Fifty-seven-year-old woman (NYHA Class II) with moderate left heart failure, oscillatory breathing, and peak 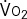 63% of predicted. The shape of the early exercise pattern is reasonable, but the highest 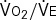 and 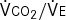 are well below normal. The rise in 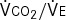 during the transition to recovery is abnormal. D, Seventy-three-year-old man (NYHA class IV) with peak 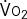 21% of predicted who died several weeks later. All values are very low. The resting, exercise, and recovery values overlay each other and on magnification can be seen to oscillate. Immediate movement to the lower left at the onset of exercise is an ominous pattern. E and F, Left heart failure in 64-year-old man before and after treatment. Raw data were obtained every 30 s rather than 10 s. In E, values move to the left (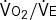 is decreasing) and upward with exercise. After treatment, peak 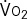 values increased from 45% to 66% of predicted. In F, resting values start higher and move upward and to the right (indicating 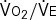 is also increasing) before moving leftward. In both studies, the highest 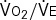 occurred when 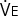 ≈21 L/min, at which time 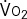 was 25×21=≈520 mL/min (E) and 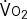 was 370×21=≈780 mL/min (F). Thus, this 50% increase in 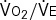 from E to F was primarily due to increased perfusion, not increased ventilation.

Six studies of successive 90-second averages of 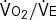
–versus–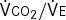 are displayed: 2 healthy subjects (Figure 1A and B), 2 patients with heart failure (Figure 1C and D), and 1 patient^23^ with heart failure before and after a change in treatment (Figure 1E and F). These displays (and many others not presented) suggest that 90-second rolling average data are optimal for heart failure with or without oscillatory breathing and also are satisfactory for healthy subjects. The 2 healthy subjects in panels A and B of the Figure, who differ significantly in sex, age, and body size, display typical healthy 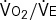
–versus–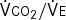 patterns. The patterns of change found in all other healthy subjects are quite similar. Panels C and D of the Figure show 2 patients with left heart failure. Panel C shows a patient with NYHA Class II heart failure with oscillatory breathing, peak 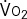 63% of predicted, and minimal 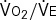 increase before a decline due to acidemia. The patient with NYHA Class IV heart failure depicted in panel D had more severe heart failure (peak 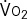 21% of predicted) and died a few weeks after this test. With very severe left heart failure, the resting values of 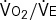 and 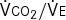 are both very low. Because the 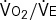 declines very soon after exercise starts in such patients, there is no appreciable movement to the northeast. Presumably, in such patients, arterial − mixed venous O_2_ blood content differences at rest already may be abnormally high, and cardiac output is unable to increase much more. Panels E and F of the Figure show a patient with moderately severe left heart failure (NYHA Class III) before and 3 months after adjustment of his drug therapy.^23^ Note the changes in resting and exercise values and the changes in pattern of 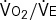
–versus–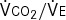 after improved therapy. A review of the raw data (not given) shows that 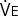 at the highest 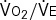 is similar in both studies, at 21 L/min. Still, at those times, the 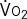 in milliliters per minute is ≈50% higher (780/520) than in panel E before therapy was improved.

Thus, in groups and individuals, both highest 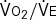 and highest 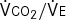 values tend to be lower in heart failure and displaced to the lower left of the figures nearer the origin. With very severe heart failure, the 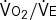
–versus–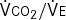 may move trivially or not at all northeastward and move immediately westward, indicating extremely limited perfusion.

## Discussion

### Advantages of 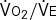
–Versus–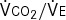


Exercise tests with gas exchange measurements have been found to assess severity in heart failure better than several more invasive procedures.^1–7,10–14,17–19^ The new display plus the data and analyses of this study (Table 3 and Table 4) reinforce the superiority of this type of test. Displaying simultaneous 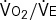
–versus–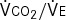 on equal X–Y axes reveals the following: (1) The highest values of 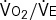 and 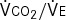, which indicate optimal matching of perfusion to ventilation, normally occur during moderate, not maximal activity. (2) The values of 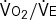 and 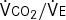 independently discriminate heart failure severity better than echocardiography, resting hemodynamics, and other exercise measurements. (3) Reductions in heart failure values from their higher reference values result primarily from inability to adequately increase cardiac output rather than from overventilation. (4) The inability to increase 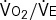
–versus–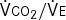 at the onset of exercise indicates very severe heart failure.

### Factors Moving Data Points

Increases or decreases in the numerators (cardiac output times blood differences) or denominators (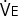) change the value and position of 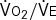
–versus–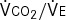 data points. Basically, 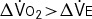 goes easterly, 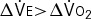 goes westerly, 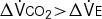 goes northerly, and 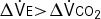 goes southerly. In healthy subjects and in those with heart failure, both C(a−v)O_2_ and C(v−a)CO_2_ differences progressively widen soon after the onset of exercise.^22,24^ In healthy subjects, after considerable variability of 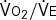
–versus–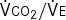 during rest, the data points usually move as follows: (1) northeasterly when cardiac output times are reasonably similar and C(a−v)O_2_ and C(v−a)CO_2_ differences increase proportionally considerably more than 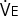; (2) more northerly as C(v−a)CO_2_ and 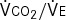 increase more than C(a−v)O_2_ and 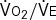; (3) sometimes briefly northwesterly when 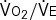 begins its decline (near the anaerobic threshold); (4) westerly or west-southwesterly when 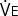 is increasing much faster than cardiac output (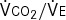 and especially 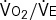 decline); (5) more southerly during early recovery when cardiac output and especially C(a−v)O_2_ decline more than 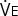 and C(v−a)CO_2_; and finally (6) although not recorded, predominantly easterly to eventually return to resting values when O_2_ debt is gradually repaid.

### Does Hyperventilation or Hypoperfusion Dominate?

It is understandable that low end-tidal and arterial CO_2_ pressures in heart failure are usually attributed to chronic hyperventilation rather than chronic hypoperfusion,^6,10–12,15,16^ although metabolic acidosis also could play a role. One's perspective changes when the numerators and denominators are reversed. These new displays and examination of the tabular data (Table 3 and Table 4) provide evidence that insufficient or inadequate perfusion rather than excessive ventilation is likely to be the major modifier of gas exchange patterns in heart failure. In assessing the exchange of O_2_ and CO_2_ in heart failure during normal activities and exercise, the past approach has been to consider high 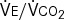, high dead space–to–tidal volume ratios, 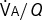 mismatching, lung restriction, and possibly metabolic acidosis and low CO_2_ set-point as the dominant factors.^4,6–8,10–12,15,16^

A high 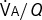 (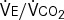) tends to be perceived differently than a low Q/VA (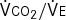). In heart failure, the factors of high 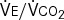, high dead space–to–tidal volume ratios, and high 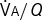 are not wrong or inaccurate, but the numerators of these ratios tend to minimize the dominant role of low perfusion in each of these ratios. Finding overly low 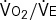
–versus–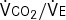 values in heart failure allows a change in perspective. When there are very low values of 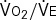
–versus–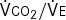 in patients with heart failure at rest, the cardiac output is likely to be reduced, with higher than normal C(a−v)O_2_ and C(v−a)CO_2_, so that further increases in the numerators during exercise are limited.

Optimally, during exercise, both perfusion and ventilation are not only “matched” but are adequate without being “wasteful.” From our perspective, perfusion and ventilation are least wasteful and best matched during moderate-intensity exercise—ie, near the anaerobic threshold (when 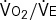 and 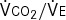 are usually highest), rather than at rest or higher-intensity exercise (when they are lower). In a normal individual during high-intensity exercise, the cardiac output, not 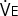, is nearly always the limiting factor. In someone with heart failure, this is even more obvious. Even though patients with heart failure have excessive dyspnea as a symptom, they are rarely ventilatory limited unless they have additional lung disease, severe lung restriction, or frank pulmonary edema. Therefore, in comparing 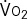 or 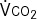 to 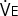, both in healthy individuals and in those with heart disease, it seems more reasonable to consider “hypoperfusion” or insufficient perfusion as the major factor causing fatigue and dyspnea rather than to consider ventilatory inefficiency, mismatching, or excessive ventilation as the major factors causing higher 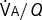, 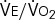, and 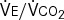 ratios, higher 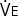
–versus–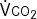 slopes, dyspnea, and fatigue.

### Limitations

(1) 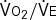
–versus–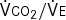 alone do not give us exact measures of cardiac output, stroke volume, C(a−v)O_2_, or C(v−a)CO_2_. Despite that, the absolute values and changing patterns of 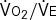
–versus–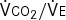 on a single X–Y plot give us new and important insights into normal physiology and the pathophysiology and severity of heart failure. (2) Other causes are likely present, but the hypothesis that low cardiac output is more important than high 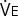 in explaining the gas exchange findings in heart failure warrants confirmation or refutation by others. (3) Because this is a preliminary study, larger numbers of healthy subjects, athletes, or patients with other heart diseases and disorders are not reported. (4) Finally, patients with anemia, primary lung diseases, or right-sided heart failure are not reported. In patients with heart failure and anemia, we would anticipate that despite reduced blood viscosity, a low exercise cardiac output would dominate. Thus, one would expect the highest 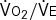 and 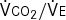 to be lower than without anemia, but we have no evidence to confirm or refute that. In patients with primary lung disease without evident primary heart disease, we have noted that some patients with severe airways obstruction may have only mild or no reductions in their highest 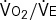 or 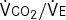 secondary to their ventilatory limitation—ie, their inability to increase ventilation normally. However, their pattern of 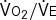
–versus–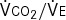 is abnormal in that they stop incremental exercise when they reach their highest values of 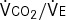 or 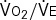, which is dissimilar to patients with exclusive left heart failure.
